# Rumen Fermentation and Fatty Acid Composition of Milk of Mid Lactating Dairy Cows Grazing Chicory and Ryegrass

**DOI:** 10.3390/ani10010169

**Published:** 2020-01-19

**Authors:** Mancoba Mangwe, Racheal Bryant, Pablo Gregorini

**Affiliations:** Faculty of Agriculture and Life Sciences, Lincoln University, P.O. Box 7647 Canterbury, New Zealand; racheal.bryant@lincoln.ac.nz (R.B.); pablo.gregorini@lincoln.ac.nz (P.G.)

**Keywords:** chicory, rumen fermentation, milk and rumen fatty acid, milk production

## Abstract

**Simple Summary:**

Enhancing the concentration of individual fatty acids (FA) in milk has been, for a long time, a major aim for researchers because certain FAs are linked with several health benefits in humans as well as improving the processing quality of milk products. It is well documented that diet, management regime, and extent of biohydrogenation in the rumen are critical in determining the composition of FA in the milk of dairy cows. This study investigated the effects of including chicory into the traditional feeding regime of ryegrass/white clover, and time of its allocation on milk production, rumen fermentation, and FA composition of milk and rumen digesta of dairy cows. Our findings show that allocation of mature chicory herbage to dairy cows at 50% of their ration modified rumen fermentation and improved both milk yield and the FA profile of the milk. Allocating chicory herbage during the afternoon is a useful strategy that can translate to improved milk production and quality. These findings reflect not just the feasibility of including chicory as part of a feeding regime, but also the role of chicory in rumen fermentation and biohydrogenation.

**Abstract:**

The goals of the current study were to investigate the effects of including chicory (*Cichorium intybus* L.) into the traditional feeding regime of ryegrass/white clover (*Lolium perenne* L./*Trifolium repens* L.), and time of its allocation on milk production, rumen fermentation, and FA composition of milk and rumen digesta of dairy cows. Nine groups of four cows were allocated one of three replicated feeding regimes: (1) ryegrass/white clover only (RGWC), (2) ryegrass/white clover + morning allocation of chicory (CHAM), and (3) ryegrass/white clover + afternoon allocation of chicory (CHPM). One cow per group had a rumen cannulae fitted. Treatment did not affect total grazing time or estimated dry matter intake, but cows ruminated more when fed RGWC than chicory. Allocating chicory in the afternoon elevated milk production compared with RGWC and CHAM. Milk from cows grazing chicory contained greater concentrations of polyunsaturated FA (PUFA) such as C18:3 c9, 12, 15 and C18:2 c9, 12 than those on RGWC. As with milk, rumen digesta concentration of PUFA increased when cows grazed on chicory rather than RGWC, which corresponded with lower concentrations of intermediate vaccenic and biohydrogenation end-product stearic acid for cows grazing on chicory. Mean ruminal pH was lower for cows offered chicory than those on RGWC, reflecting greater rumen concentrations of volatile fatty acids (VFA) for cows fed chicory. Allocating chicory during the afternoon is a useful strategy that can translate to improved milk production. The lower rumen pH, lower concentration of vaccenic and stearic acids, and elevated concentration of PUFA in the rumen of cows fed chicory suggest reduced biohydrogenation and may explain the elevated concentration of PUFA in the milk of cows fed chicory compared with those fed RGWC.

## 1. Introduction

Enhancing the concentration of individual fatty acids (FA) in milk has been, for a long time, an important aim for researchers because certain FAs are linked with health benefits in humans [1] as well as improving the processing quality of milk products. Fifty percent of the FA found in milk are sourced from the blood and the remaining 50% are synthesized in the mammary gland [2]. Those FA synthesized in the mammary gland tend to be short-chain acids (C4:0 to C14:0) and are largely influenced by animal genetics [3]. The FA sourced from blood are predominantly of diet or microbial origin, with lipolysis and the mobilization of body fat accounting for 5% in a well-fed animal to over 20% of milk FA in early lactation when cows are in a negative energy balance [4,5]. The content and composition of FA of microbial origin varies markedly, and typically represent the odd-chain and branched FAs (OBCFA). Researchers have attempted to use these milk FAs to predict volatile fatty acid (VFA) production in the rumen as a measure of diet effect on rumen function [6]. 

Milk FA derived from the diet is also variable and represents long chain polyunsaturated fatty acids (PUFA), which have been linked to several positive human-health related effects [7]. Diet FA, particularly PUFA, are extensively biohydrogenated in the rumen, which reduces their concentration in milk [8]. Plant factors can influence this process, providing opportunities to manipulate FA proportions in the rumen and thereby in the milk [9]. For example, diets high in readily fermentable carbohydrates are known to increase total VFA and reduce pH during ruminal fermentation, limiting lipolysis, and thus, biohydrogenation [10,11]. Chilliard et al. [1] reported a 35% to 50% decrease in ruminal biohydrogenation of PUFA, such as linoleic (LA; C18:2 c9, 12) and α-linolenic acid (ALA; C18:3 c9, 12, 15) when concentrates formed more than 70% of the diet, a result of reduced biohydrogenation at lower rumen pH. 

Alternative forages, such as chicory (CH; *Cichorium intybus* L.) and plantain (*Plantago lanceolata* L.) present an opportunity to improve the FA composition of milk whilst meeting environmental and economic requirements in pastoral livestock systems. Chicory has long been considered a useful component of the pastoral system in temperate regions [12], providing benefits of improved mineral nutrition [13,14] and producing a large amount of high-quality summer feed compared to RGWC when sown as monoculture or in diverse swards [15,16,17]. Chicory contains more readily fermentable carbohydrates (non-fibre carbohydrates; NFC) than ryegrass/white clover (*Lolium perenne* L./*Trifolium repens* L.; RGWC) herbage [18], which may increase the concentration of total VFA and lower rumen pH during ruminal fermentation. In our earlier proof-of-concept research, elevated PUFA were recorded from late lactation cows fed pasture diets of CH compared with the control RGWC in a grazing study [19]. Feeding CH, as all or as part of a ration, has also shown milk yield improvements [20,21] and nitrogen loss reductions [18,22,23] compared to traditional ryegrass pastures. 

However, Chapman et al. [20] pointed out the need to consider strategies to integrate alternative feeds into farm systems. Although our previous research showed that at 100% of the diet, CH could improve milk FA profile and milk production, it is not feasible to include CH at 100% of pastoral diets for extended periods. Muir et al. [24], feeding partial mixed ration demonstrated the feasibility of feeding CH at 50% and increasing milk PUFA. To capture the value of alternative forages as a means for improving product quality in terms of milk FA, more information is required to understand the mechanisms leading to increased PUFA from CH diets and associated feeding management. In a review, Gregorini [25] showed that a small change from the routine allocation of fresh herbage of one forage species could have positive benefits on animal performance and environmental impact. Indeed, Abrahamse et al. [26] demonstrated that allocating pasture to dairy cows in the afternoon compared with the morning altered milk composition and fat yield. 

To better understand the rumen fermentation factors influencing milk FA profile of CH herbage and identify suitable feeding regimes to capture forage derived benefits on milk quality, a grazing study was conducted comparing rumen fermentation and fatty acid composition of rumen and milk of mid-lactating dairy cows on CH or conventional RGWC pastures.

## 2. Materials and Methods 

### 2.1. Experimental Site and Design

The experiment took place between 10 December 2018 and 27 January 2019 at the Lincoln University Research Dairy Farm, about 20 km south of Christchurch in Canterbury, New Zealand (43°38′ S, 172°28′ E; 17 m above sea level) with the approval of the Lincoln University Animal Ethics Committee (AEC #2018-48). The experiment was organized in a completely randomized design with three replicated feeding regimes: (1) perennial ryegrass/white clover only (RGWC), (2) ryegrass/white clover + morning allocation of chicory (CHAM) and (3) ryegrass/white clover + afternoon allocation of chicory (CHPM). 

The pastures used in this experiment were second-year CH and sixth-year RGWC. Details of establishment were given in Mangwe et al. [21]. Briefly, the ryegrass (cv. Arrow AR1; 20 kg/ha) and white clover (cv. Weka; 3 kg/ha) swards were established in October 2013 while CH (cv. Choice, 5.3 kg seed/ha) swards were established in November 2017 following cultivation. The soil was classified as free-draining Templeton fine sandy loam soil (Hewitt 2010) with a soil pH of 6.2 (1: 2.1 *v/v* soil–water slurry), Olsen phosphorus of 29.7 mg/L, potassium of 0.9, calcium of 8.2, magnesium of 1.1, and sodium of 0.2 me/100 g as determined on 29 September 2017 to 75 mm depth. We did not apply any fertilizer during establishment. For the current research, the experimental area of 10.5 ha consisting of 7 × 1.5 ha paddocks was prepared three to four weeks prior to the study. To ensure that all plants had accumulated similar growing degree days during the experiment and to build a feed wedge, a third of each paddock was rotationally grazed using a group of cows, and mowing after grazing to a uniform height of 4 cm. Nitrogen fertilizer was applied at 30 kg N/ha as urea immediately after grazing each paddock.

### 2.2. Animals and Management

The experiment included a 4-week baseline measurement period, where all cows grazed RGWC plus 15–20% of the diet as CH herbage daily, a 6-day adaptation period in which the relative proportion of the diet was increased to 50% of the diet, and a 12-day measurement period. Based on results obtained during the baseline measurement period, 36 mid-lactating Friesian × Jersey dairy cows on their second to fourth parities were stratified into nine groups of four cows and assigned to one of the three replicated (*n* = 3) feeding regimes (RGWC, CHAM, and CHPM). One cow per group had a rumen cannulae fitted (Bar-Diamond; Parma, Idaho, USA). Cows were stratified according to (mean ± standard error of the mean; SEM)); milk fat content (5.08 ± 0.25 g/100 g of milk), milk protein content (3.78 ± 0.06 g/100 g of milk), milk solid yield (MS; 1.82 ± 0.08 kg/cow per d), milk yield (21.3 ± 0.97 kg/cow per d), days in milk (155 ± 3.3 days), and live body weight (483 ± 13.8 kg).

**Figure 1:**
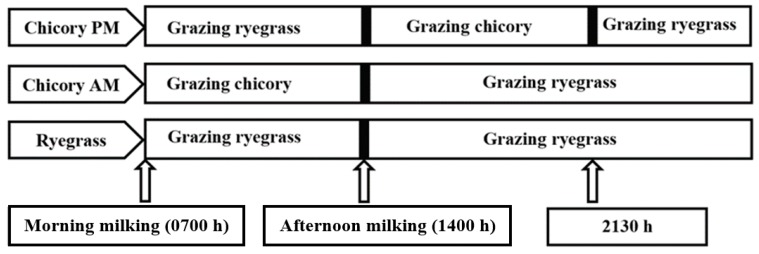
Management regimes during the experiment. Control cows offered ryegrass only (RGWC), received a fresh allocation after 24 h following the morning milking (around 0800 h). Cows on Chicory AM received a new allocation of chicory herbage between morning and afternoon milking (0800 h and 1330 h) and a new allocation of ryegrass herbage following afternoon milking. Cows offered Chicory PM received a new allocation of ryegrass herbage between morning and afternoon milking, a new allocation of chicory herbage following afternoon milking (1530 h–2100 h), after which they went back to their previous ryegrass allocation.

Both CH and RGWC herbages were grazed in situ using similar herbage allowance. Target allowance was 34 kg of dry matter (DM) per cow per day above ground level to maintain baseline milk production. Allocations were based on herbage mass determined every three days by harvesting to ground level herbage within three 0.25-m^2^ quadrat cuts per break, and weighing the washed, dried material. Details of the management regimes during the experiment are summarized in Figure 1. Briefly, control cows offered RGWC received a fresh allocation (34 kg DM/cow per day above ground) after 24 h, following the morning milking. Cows offered either of the CH treatments were allowed to graze CH for five and a half hours before returning to RGWC. The cows on CHAM received a new allocation of CH herbage (17 kg DM/cow per day) between morning and afternoon milking (0800 and 1330 h) and a new allocation of RGWC herbage (17 kg DM/cow per day) following afternoon milking. Cows offered CHPM received a new allocation of RGWC herbage (17 kg DM/cow per day) between morning and afternoon milking, a new allocation of CH herbage (17 kg DM/cow day) following afternoon milking (1600–2130 h), after which they went back to their previous RGWC allocation. Temporary fencing was used to control cows. All cows had free access to fresh water at all times. 

### 2.3. Herbage Measurements

Representative herbage samples for chemical and botanical composition were harvested to ground level from each of the mornings and the afternoons’ allocations preceding the cows moving into their allocations (0700 h and 1300 h, respectively) on day 6, 8, 11, 16, and 18 of the experiment. Samples were transported to the research facilities, homogenized, and sub-sampled for botanical and chemical analysis. Each sub-sample was separated into sown species, reproductive, vegetative, weed, and dead material. The separated components were dried at 60 °C for at least 48 h. The DM content of the homogenized sub-sample was determined by immediately recording fresh weight and dry weight after oven drying at 60 °C for 48 h. The remaining homogenized fresh herbage sample was freeze-dried and ground to pass a 1-mm sieve (ZM200 Retsch). Organic matter (OM), water-soluble carbohydrates (WSC), neutral and acid detergent fibre (NDF, ADF), crude protein (CP), dry matter digestibility (DMD), organic matter digestibility (OMD), and digestible organic matter in the dry matter (DOMD) from the dried ground samples were estimated using near-infrared spectrophotometry (NIRS, Model: FOSS NIRSystems 5000, Maryland, USA). The NIRS calibration for WSC [27], crude protein (Variomax CN Analyser, Elementar), NDF [28] and ADF (method 973.18; AOAC, 2012), DOMD, and DMD [29] were previously derived from RGWC and CH forages. All R-squares for predicting the nutrients measured were similar and were above 0.9. All samples were well within the calibration range. 

Total dry matter intake (DMI) was estimated on day 6, 9, 11, 16, and 18 of the experiment as the sum of CH and RGWC herbage apparent intakes. Apparent intake was estimated using the formula; intake (kg DM/cow/day) = (((pre kg DM ha^−1^ − post kg DM ha^−1^) ÷ No. cows) × area). Pre-graze and post-graze were based on herbage harvested to ground level within three 0.25-m^2^ quadrats before and after grazing respectively.

### 2.4. Milk Yield and Composition Measurements 

Milk yield was recorded daily at 0700 and 1400 h with an automated system (DeLaval Alpro Herd Management System, DeLaval, Tumba, Sweden). Individual cows’ milk was sampled on days 6, 8, 11, 16, and 18 of the experiment for further analysis. Milk fat, protein, and lactose contents were determined from fresh milk using Milkoscan™ (Foss Electric, Hilleroed, Denmark). Milk samples for FA composition were taken from individual cows on day 16 and 18 of the experiment. 

### 2.5. Grazing Behaviour

Three cows in each group were fitted with SensOor ear-tags (Agis Ltd, the Netherlands) to continuously record time spent grazing, ruminating and idling per day.

### 2.6. Rumen Sampling

Rumen fluid and digesta samples were collected from the nine ruminal cannulated cows at four-hourly intervals starting from 0400 to 2400 h on day 13 and 15 of the experiment. Rumen digesta samples were collected by hand, via the rumen cannulae, from the mid and dorsal rumen where fermentation is most active. Rumen fluid samples for determination of VFA were attained by squeezing a subsample of the composited rumen digesta through two layers of cheesecloth and stored at −20 °C pending analysis. Volatile fatty acids concentration from rumen fluid samples were determined using a Gas Chromatograph (GC: Shimadzu GC-2010, Kyoto, Japan) fitted with an SGE BP21 30 m × 530 μm × 1.0 μm wide-bore capillary column. The remaining rumen digesta sample was mixed, subsampled, placed in resealable plastic bags and immediately stored at −20 °C pending FA analysis. Each of the nine cows had intra-ruminal smaXtec pH sensors (Smart Farm Data Limited, New Zealand) inserted before the experiment to continuously measure pH, but software failure resulted in an incomplete data set. Ruminal pH was therefore measured from thawed rumen fluid samples taken at four-hourly intervals, using pH probe (HD 2105.2 pH/mv meter; Delta Ohm Inc., Padua, Italy). Samples were kept chilled during pH measurements. 

Rumen digesta, herbage and individual cow milk samples for FA acid were prepared by transmethylation and analyzed by gas chromatography (with AOC-20i auto-sampler, Shimadzu GC-2010, Japan), according to AOAC (2012) Method 2012.13 using a Varian CP742 silica capillary column (0.25 × 100 m × 0.2 µm).

### 2.7. Statistics and Calculations

For all analyses, we used a mixed-effects model in R. The animal group (paddock) was used as an experimental unit. For data taken from all cows (milk yield, composition, and FA profile), treatment (CHAM, CHPM, and RGWC) was included as a fixed effect, while animal nested in sampling day used as random effect. For data taken at paddock level (herbage composition and intake), treatment and forage type (CH and RGWC) and their interaction were included as fixed terms while day used as random effect. To explore diurnal patterns in rumen fermentation parameters and rumen FA composition, treatment was included as fixed effect, sampling time (0400, 0800, 1200, 1600, 2000, 2400 h) as a repeated measure, while animal nested on sampling day used as random effect. For all data, means separation was done using the ‘emmeans’ package of R, with Tukey’s method for comparing the estimates. A significant difference was declared at *p* < 0.05, while a tendency was declared at *p* < 0.10.

## 3. Results

### 3.1. Herbage Characteristics

In CH pastures, mean CH herbage accounted for an average of 821 ± 21 g/kg of the biomass, while ryegrass accounted for 612 ± 32 g/kg on a dry weight basis in RGWC pastures. Chicory swards were at reproductive stage, with the reproductive stem accounting for an average of 394 ± 14 g/kg DM of the CH herbage. The reproductive stem in ryegrass herbage accounted for 121 ± 5.9 g/kg of DM. Chicory swards had less than 50 g/kg white clover, while ryegrass swards had 93 ± 8.7 g/kg of DM white clover. In RGWC swards, dead material accounted for 192 ± 11 g/kg and weed content accounted for 114 ± 7.8 g/kg of DM. The corresponding proportions of dead material and weed content were less than 50 and 101 ± 6.6 g/kg of DM, respectively, on CH pastures.

Herbage mass and pre-grazing chemical composition are presented in Table 1. Treatment did not affect pre- and post-graze mass. However, CH herbage was grazed to a lower residual height than RGWC herbage (1387 kg/ha DM vs. 1700 kg/ha DM; *p* < 0.001). Water-soluble carbohydrates, ADF, crude fat and digestibility were similar for all treatments, whereas CP, NDF, and NFC differed between treatments. There was an interaction between herbage type and time of allocation for DM, NDF, DOMD, and NFC (*p* < 0.05). Generally, CH herbage had greater NFC, but less DM, OM, CP, NDF, and ADF contents than RGWC (*p* < 0.05). Herbage offered in the afternoon had greater concentrations of DM, NFC, and DOMD than herbage allocated in the morning, regardless of herbage type. 

Time of allocation did not affect total diet FA (Table 1; *p* > 0.05), but CH herbage had a greater concentration of total FA than RGWC herbage (24.3 vs. 18.4 ± 1.89 mg/g DM). The predominant FAs in the herbage were LA and ALA, which accounted for 27.1 and 47.9%, respectively in CH and 16.9 and 57.6% in RGWC herbage, respectively. 

The reproductive stem in the herbage after grazing doubled, highlighting selection against these plant components by cows as stem accounted for an average of 703 ± 24 g/kg DM in CH swards and 225 ± 6.2 g/kg DM in RGWC swards. Post grazed CH herbage had 389, 103, and 635 g/kg, NDF, CP, and DOMD, while RGWC herbage had 559, 110, and 634 g/kg, NDF, CP, and DOMD, respectively. The complete chemical composition of post grazing residuals is presented in Table S1. 

### 3.2. Dry Matter Intake and Grazing Behaviour

**Figure 2:**
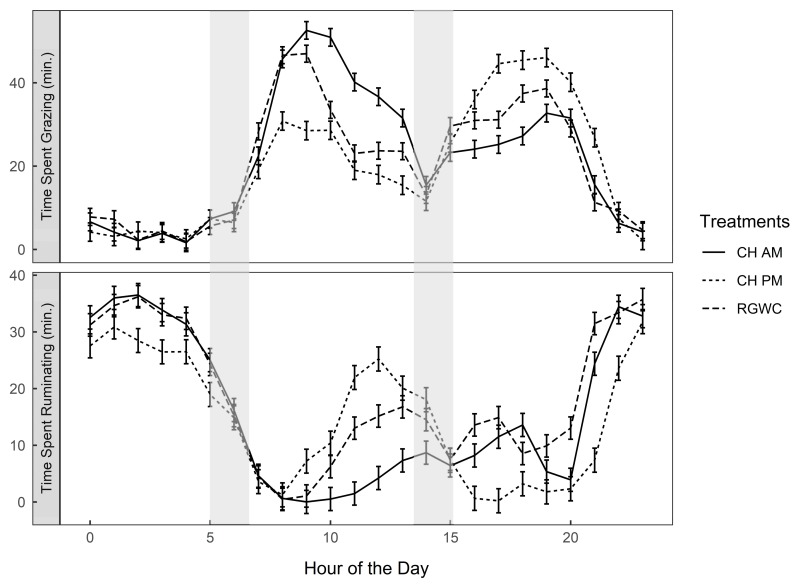
Diurnal variation of time spent grazing and time spent ruminating (min/h). Solid lines denote CHAM (ryegrass/white clover + morning allocation of chicory), short dashed line denotes CHPM (ryegrass/white clover + afternoon allocation of chicory), and long dashed line denote RGWC (perennial ryegrass/white clover only). Shaded areas represent AM and PM milking events. Error bars are standard error of the mean. Abbreviation; CH, chicory.

Feeding regime did not affect apparent DMI (Table 2). On average, CH accounted for 55% and 58% of total DMI for cows offered the CHAM and CHPM, respectively. Total time spent grazing per day was also unaffected by feeding regime (499 ± 15 min/cow per day; *p* = 0.167), but cows offered RGWC spent more time ruminating (446 ± 9.34 min/cow per day) than those offered CHAM (379 ± 9.69 min/cow per day) or CHPM (359 ± 10.13 min/cow per day; *p* < 0.0001). Irrespective of treatment, cows consumed the majority of forage during two major grazing bouts (0900–1300 h and 1600–2000 h; Figure 2). When cows were grazing CH, the intensity (min/h) and duration of their grazing was greater than on RGWC. Cows offered CHAM grazed more intensely in the morning, spending nearly 211 vs. 109 and 150 min/5 h between 0900–1300 h compared with CHPM and RGWC, respectively. Whereas cows offered CHPM grazed more intensely during the afternoon, spending 212 min vs. 140 and 167 between 1600–2000 h compared with CHAM and RGWC, respectively. 

### 3.3. Milk Production and Composition 

When compared with RGWC, including CH increased milk production (Table 2). Although CHPM and CHAM had similar milk yield, elevated milk fat percent (*p* = 0.051) and milk fat yield for CHPM resulted in greater milk solids than CHAM. Milk protein percent was increased in cows offered the CHAM compared with those offered the CHPM or RGWC.

Cows on CHAM and CHPM feeding regimes had milk with similar concentrations of LA and ALA, but greater than the RGWC (Table 3). Cows on RGWC increased vaccenic acid (VA; C18:1 t11) compared with the CHAM or CHPM regimes. Conjugated linoleic acid (c9, t11 C18:2; CLA) was similar for cows on CHAM and RGWC, but tended to be greater for those on RGWC than those on CHPM (*p* = 0.05). Odd and branched-chain FA such as isoC15:0, anteisoC15:0, C17:0, and isoC17:0 were greater in cows fed RGWC than those fed CH. The sum of saturated fatty acids was not affected by feeding regime (*p* = 0.60), but CH inclusion increased the concentration of PUFA in the milk, regardless of the time of allocation (*p* < 0.0001). 

### 3.4. Rumen Fermentation Parameters

**Figure 3:**
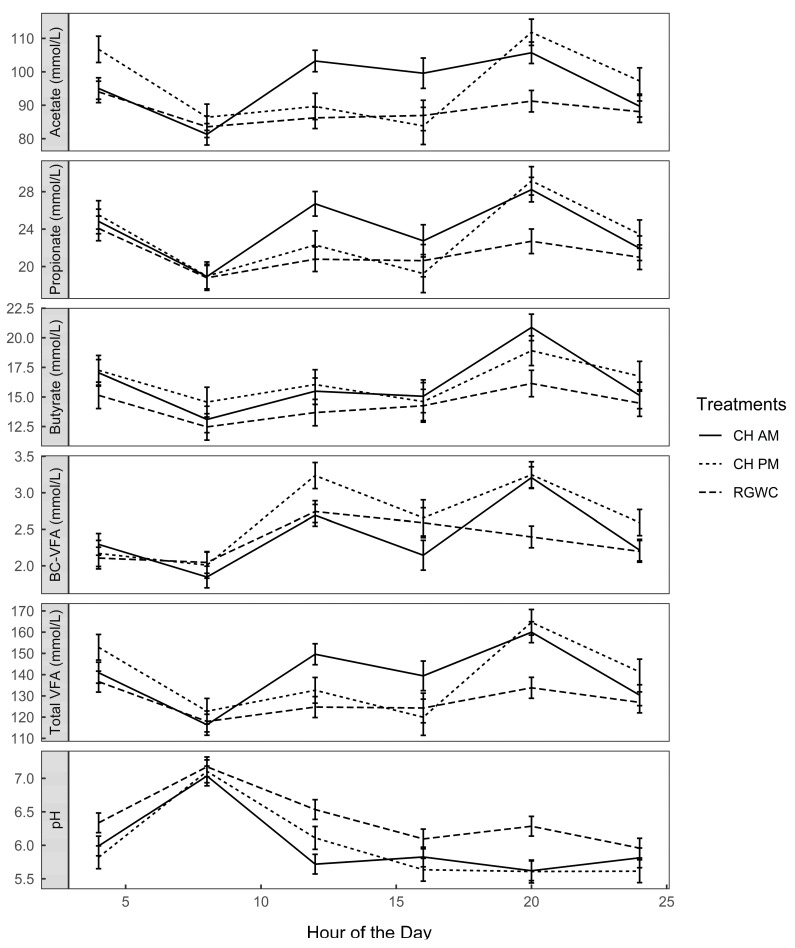
Diurnal variation of rumen fermentation parameters. Solid lines denote CHAM (ryegrass/white clover + morning allocation of chicory), short dashed line denotes CHPM (ryegrass/white clover + afternoon allocation of chicory), and long dashed line denote RGWC (perennial ryegrass/white clover only). BC-VFA (branched chain-volatile fatty acids; iso-valerate + iso-butyrate). Error bars are standard error of the mean (*n* = 3).

Major VFAs (acetic, propionic and butyric acid) accounted for nearly 97% of total VFA. The four-hourly diurnal variations in the individual VFAs demonstrate the time × treatment interaction and reflect the variation in feeding patterns of the different regimes (Figure 3). However, on average cows offered CH (CHAM and CHPM) had greater total concentrations of VFA than animals grazing RGWC (140 *vs.* 128 ± 4.3 mmol/L; *p* < 0.0001). 

Fluctuations in VFA profiles corresponded with changes in rumen pH (Figure 3). For all feeding regimes, ruminal pH was highest (pH = 7.1 ± 0.17) at the end of the allocation period between 0400 and 0800 h. Rumen samples taken at 1200 h, four hours after the morning allocation of fresh herbage, indicated significant reductions in pH of all treatments. However, pH was more reduced for cows on CHAM (pH = 5.72 ± 0.15), intermediate for cows on CHPM (pH = 6.11 ± 0.17) and least affected for cows on RGWC (pH = 6.53 ± 0.15). The complete VFA concentration in ruminal contents in cannulated cows is presented in Table S2.

### 3.5. Rumen Long-Chain FA Composition

**Figure 4:**
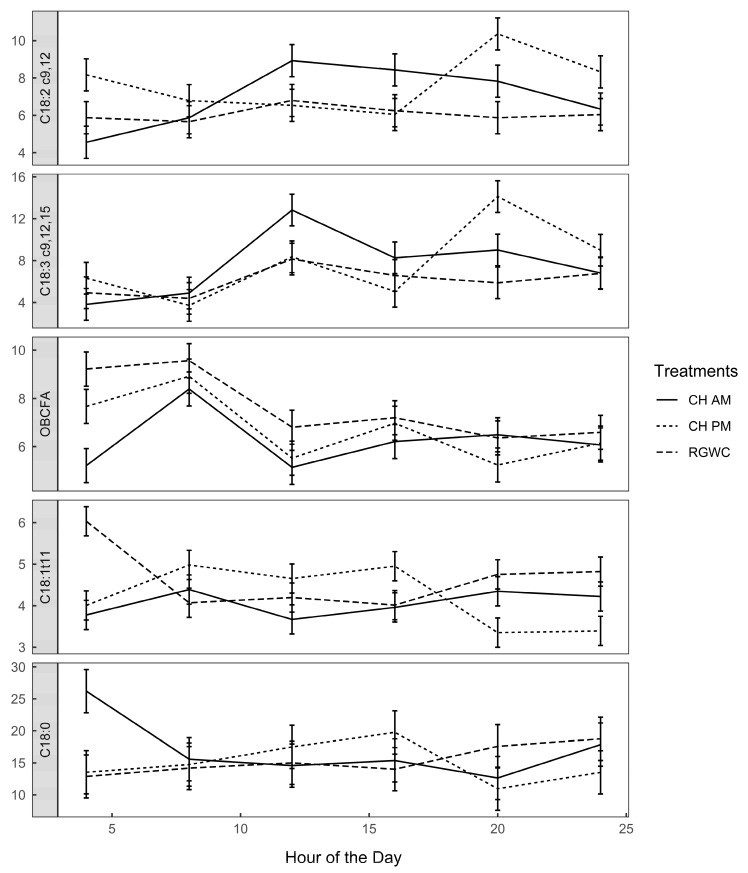
Diurnal variation of rumen fatty acids (g/100 g of total FA). Solid lines denote CHAM (ryegrass/white clover + morning allocation of chicory), short dashed line denotes CHPM (ryegrass/white clover + afternoon allocation of chicory), and long dashed line denote RGWC (perennial ryegrass/white clover only). Error bars are standard error of the mean (*n* = 3).

The mean concentration of LA in the rumen was 6.99, 7.71, and 6.08 ± 0.45 g/100 g of total FA, while that of ALA was 7.61, 7.76, and 6.11 ± 1.1 g/100 g of total FA for cows on CHAM, CHPM, and RGWC feeding regimes, respectively. Diurnal patterns of selected rumen FA are depicted in Figure 4. There was a significant feeding regime × sampling time interaction for LA, ALA, VA, and stearic acid (C18:0). Rumen LA and ALA concentrations were 34% and 56% greater for cows on CHAM than cows on RGWC or CHPM at 1200 h. Cows on CHPM had 90% greater rumen ALA at 2000 h compared with cows on RGWC or CHAM. The increase in these plants derived PUFA corresponded with a sharp decline in biohydrogenation intermediate VA and biohydrogenation end-product stearic acid. The complete list of rumen FA is presented in Tables S3 and S4.

## 4. Discussion

This is the second study in a series of experiments investigating the effect of high moisture forages on the production and quality of milk and associated environmental impacts in pastoral dairy systems. Our first proof-of-concept study showed that under pastoral grazing feeding high moisture, herb diets altered milk FA composition without affecting production [19]. The results of the present study confirm the positive effect of feeding CH on PUFA and demonstrate that producers can influence FA composition through changes in feeding regimes. 

### 4.1. Milk Production and Rumen Fermentation

It is interesting that feeding reproductive CH at 55–58% of the diet of mid lactating dairy cows increased milk production compared to the control-feeding regime, a result similar to previous findings when CH was fed while vegetative [19,20]. Muir et al. [30], on the other hand, did not observe any differences in milk production between the control ryegrass and reproductive CH at 50% of the diet in summer. In their experiment, Muir et al. [30] attributed the lack of milk production response of cows fed CH compared with RGWC on the stem material, which influenced DM, NDF, and metabolizable energy; and therefore, intake and milk production responses. Farmers may need to consider refusal of stems in feed allocation of second year chicory to avoid underfeeding. In the current experiment, total DMI did not differ between treatments, suggesting that the differences in milk response observed are explained by other factors such as grazing behavior, forage utilization, and/or nutritive value of the forages. 

Cows increased their grazing intensity during the CH feeding periods (Figure 2). Chicory herbage consisted of 39% reproductive stem before grazing and 70% of reproductive stem after grazing, suggesting that the cows selected leaf over stem material. Clark et al. [31] reported enhanced animal performance at high leaf allowances from reproductive CH swards, a result similar to our experiment. The ratio of non-structural to structural carbohydrates was also greater for CH compared to RGWC herbage, which might have improved energy supply. Ruminants get nearly 70–80% of their energy supply from VFAs [32]. The mean concentration of total VFA was 10% greater for cows on CHAM and CHPM than those on RGWC were, which likely explains their increased milk production.

When comparing the two CH treatments, afternoon allocation of CH increased milk solid yield by 7.6% compared with the morning allocation. This reflects the greater fat percent (*p* = 0.051) and daily fat yield (*p* < 0.001) from cows offered CH in the afternoon than those offered CH in the morning and can be explained by the increased proportion of branched chain-VFA (iso-butyrate + iso-valerate) in the rumen of CHPM cows than CHAM cows (Figure 3). Increased proportion of branched chain-VFA in the rumen is associated with improved milk FA composition and milk yield in dairy cows [33]. Although studies have looked into the timing of fresh herbage allocation and its effect on milk production [26,34,35], this is the first experiment to demonstrate the impact of timing of allocation of two different forage species on milk FA composition. The findings from the current study suggest that cows are more responsive to timing of allocation of herbage of some species (CH) more than others (RGWC) on milk yield and milk composition. 

Another interesting observation arose from the evident synchrony between grazing behavior and rumen fermentation. Rumen VFAs, especially acetate and propionate, increased during peak grazing periods, with greater concentrations occurring when cows grazed CH than RGWC (Figure 3). The increase in the concentration of total VFA in rumen corresponded with declines in rumen pH in all treatment cows. pH values less than 5.8 are regarded as harmful to ruminal cellulolytic bacteria [36], whereas pH less than 5.5 is said to be detrimental to the ruminal epithelium and VFA absorption in cows fed a high-concentrate diet [37]. Cows fed RGWC were able to maintain their rumen pH above 5.8 likely because they ruminated more. Rumination increases saliva production rate and increases the supply of bicarbonate to the rumen to enhance total ruminal buffering capacity [38]. Although ruminal pH was below 5.8 in cows offered CH between 1200 h and 2000 h in the current experiment, they were within a normal range of 5.6 to 6.4 previously reported in a review of 23 studies for dairy cows fed high-quality herbage [11]. High-quality herbages are highly digestible and their ruminal fermentation is associated with increased VFA, but low lactic acid [39]. Since VFAs provide most of the energy requirement of ruminants, it is not surprising that cows fed high-quality herbage diets produce more milk even at lower pH values [38].

### 4.2. Milk and Rumen Digesta FA Composition

Our results confirm that, regardless of time of allocation, feeding CH at up to half the ration increases concentrations of beneficial FA; LA and ALA, in the milk of dairy cows compared with the traditional feeding regime of RGWC [19,24,30]. The increase in the concentration of these is particularly important given their human health benefits. Alpha linolenic acid for example, has demonstrated potential to exert neuroprotective, anti-inflammatory, and antidepressant properties [40,41]. de Goede [42] recently reported that increased ALA intake lowered the risk of stroke. In the body, ALA is converted to eicosapentaenoic acid, a FA that is known for its cardio-protective and other human health benefits [43]. The concentration of these LA and ALA in milk mainly depends on their concentration in the diet and intake, level of biohydrogenation in the rumen, and amount absorbed in the duodenum [44]. The higher concentration of LA in CH diets, compared with RGWC, is likely to explain its elevated concentration in the milk, though other mechanisms are also likely to be involved in the increased milk ALA concentrations, as concentrations in herbage were not different to the control. Mean concentrations of LA and ALA were 21% and 26% greater in the rumen digesta of cows grazing CH than those on RGWC, respectively, with peak concentrations occurring four hours after allocation of CH herbage (Figure 4). The greater concentration of LA and ALA in rumen digesta of cows grazing CH corresponded with lower concentration of VA and stearic acid. This suggests that the level of biohydrogenation was reduced when cows grazed CH, which increased their rumen outflow and subsequently their inter alia availability in the mammary gland, a similar premise shared by Szczechowiak et al. [45] in milk of cows, fed condensed tannins and fish-soybean oil blend mixture. 

There are two plausible explanations for the decreased biohydrogenation when cows grazed CH, with the first being due to lower pH. Lower ruminal pH is known to inhibit the activity of lipase thus limiting lipolysis [46]. Given that lipolysis is a prerequisite for ruminal biohydrogenation, it is not surprising that more PUFA were recovered in milk of cows grazing CH. The other likely reason could be a faster rumen passage rate as a result of reductions in microbial contact with dietary FA [44,47]. This premise is supported by the decreased concentration of odd and branched-chain FA in the rumen (Figure 4) and milk (Table 3) of cows grazing CH-based diets, as their lower concentrations in the rumen and milk of cows on CH indicate reduced microbial colonization of CH than RGWC herbage. 

Milk from cows on RGWC had greater concentrations of VA (22% higher; *p* = 0.007) than cows on CHAM or CHPM (Table 2). Similarly, the RGWC regime elevated the concentration of CLA by 17% (*p* = 0.24) and 26% (*p* = 0.059) compared to cows on CHAM and CHPM feeding regimes, respectively. About 70% to 90% of CLA in the milk of cows originates from the oxidation of the precursor VA in the mammary gland and other tissues by enzyme ^Δ^9 desaturase [48], hence, the strong relationship between the VA and CLA in cow milk. The concentration of VA was 2.6, 2.79, and 2.75 times that of CLA for cows grazing CHAM, CHPM, and RGWC, respectively in the current study, which is a little greater than the 2 to 2.5 reported by Elgersma and Tamminga [49].

## 5. Conclusions

Allocation of mature CH herbage to dairy cows at 50% of their ration improved both milk yield and the FA profile of the milk. Furthermore, offering CH in the afternoon compared with that in the morning increased the milk concentration and yield of desirable polyunsaturated fatty acids. Changes in milk yield were associated with increased utilization of high-quality leaf components of CH herbage compared with RGWC herbage. While changes in milk FA composition related to CH feeding, appear to be linked to reduced biohydrogenation of dietary FA, at lower rumen pH, which subsequently increased their concentration in milk of cows fed CH. Allocating CH herbage during the afternoon is a useful strategy that can translate to improved milk production and quality.

## Figures and Tables

**Table 1:** Herbage mass, pre-grazing chemical composition and fatty acid (FA) profile of chicory and ryegrass/white clover (RGWC) sampled to ground level.

Herbage	Chicory Herbage		Ryegrass/White Clover Herbage			SEM ^5^	*p*-Value		
Treatments	CHAM ^1^	CHPM ^1^	CHAM ^1^	CHPM ^1^	RGWC ^1^		Treatment	Herbage	T x H
Pre-graze mass (kg/ha DM)	2985	3150	2958	2960	3277	175	0.375	0.541	0.645
Post-graze mass (kg/ha DM)	1372	1402	1670	1740	1687	83	0.282	<0.001	0.812
Organic matter (g/kg DM)	868	881	922	921	918	5.1	<0.001	<0.001	0.152
Dry matter (g/kg DM)	119	135	220	194	212	5.4	<0.001	<0.001	0.004
Water soluble carbohydrates (g/kg DM)	145	197	233	210	206	7.8	0.196	0.011	0.075
Crude protein (g/kg DM)	146	127	157	153	165	6.4	0.024	0.014	0.293
Neutral detergent fibre (g/kg DM)	296	246	446	473	455	9.4	<0.001	<0.001	<0.001
Acid detergent fibre (g/kg DM)	230	214	263	268	262	4.2	0.1	<0.001	0.191
Dry matter digestibility (g/kg DM)	782	798	743	736	748	5.2	0.373	<0.001	0.281
DOMD ^2^ (g/kg DM)	710	757	737	706	729	10.4	0.579	0.112	<0.001
Crude fat (g/kg DM)	38.8	41.8	41.8	45.1	42.6	0.8	0.248	0.099	0.942
NFC ^3^ (g/kg DM)	401	454	277	250	256	11.5	<0.001	<0.001	0.002
**Fatty acids (mg/g DM)**									
C14:0	0.06	0.062	0.10	0.09	0.08	0.01	0.501	<0.001	0.503
C16:0	4.76	4.62	3.51	3.35	3.14	0.3	0.034	<0.001	0.968
C18:0	0.23	0.23	0.32	0.31	0.29	0.02	0.562	<0.001	0.692
C18:1 c9	0.39	0.39	0.39	0.38	0.41	0.03	0.844	0.902	0.921
C18:2 c9,12	6.71	6.48	3.23	3.13	2.99	0.38	<0.001	<0.001	0.864
C18:3 c9,12,15	11.1	12.2	10.9	10.8	10.1	1.25	0.438	0.295	0.395
Saturated FA	5.49	5.34	4.33	4.14	3.91	0.33	0.056	0.002	0.968
Monounsaturated FA	0.53	0.53	0.61	0.58	0.58	0.46	0.86	0.20	0.81
Polyunsaturated FA	18.8	17.7	14	14.1	13.1	1.56	0.137	0.005	0.471
Others ^4^	0.65	0.62	0.66	0.64	0.610	0.03	0.530	0.705	0.869
Total FA	24.9	23.6	19	18.8	17.5	1.89	0.122	0.005	0.561

^1^ RGWC = perennial ryegrass/white clover only; CHAM = ryegrass/white clover + morning allocation of chicory; CHPM = ryegrass/white clover + afternoon allocation of chicory. ^2^ DOMD = Digestibility of the organic matter in the dry matter. ^3^ NFC = Non-fibre carbohydrates (1000- (NDF + CP + Fat + Ash)). ^4^ Others = C15:0; C16:1 c9; C17:0; C18:1 c11; C20:0; C20:2 c11,14; C20:3 c11,14,17; C22:0; C23:0; C24:0. ^5^ SEM = standard error of the mean.

**Table 2:** Estimated dry matter intake (DMI; kg/cow per day of DM), milk yield and milk composition from cows fed grazing CHAM, CHPM, and RGWC.

Item	CHAM ^1^	CHPM ^1^	RGWC ^1^	SEM ^2^	*p*-Value
Chicory intake	9.00	9.31	-	0.57	0.578
Ryegrass intake	7.34b	6.81b	16.6a	0.23	<0.0001
Total DMI	16.3	16.1	16.6	0.25	0.122
Milk yield (kg/cow per day)	21.0ab	22.0a	19.9b	0.43	<0.0001
Milk solids (kg/day)	1.84b	1.96a	1.71c	0.03	<0.0001
Fat (g/100 g of milk)	4.95	5.14	4.92	0.10	0.051
Protein (g/100 g of milk)	3.85a	3.75b	3.71b	0.03	0.002
Lactose (g/100 g of milk)	5.06	5.05	5.07	0.02	0.662
Protein: Fat	0.79a	0.74b	0.76b	0.01	0.004
Fat yield (kg/day)	1.04b	1.13a	0.97b	0.02	<0.0001
Protein yield (kg/day)	0.82a	0.83a	0.74b	0.02	<0.0001
Lactose yield (kg/day)	1.06ab	1.11a	1.01b	0.02	<0.0001

a–c Means within a row with different letters differ (*p* < 0.05). ^1^ RGWC = perennial ryegrass/white clover only; ^1^ CHAM = ryegrass/white clover + morning allocation of chicory; CHPM = ryegrass/white clover + afternoon allocation of chicory. ^2^ SEM = standard error of the mean.

**Table 3:** Milk fatty acid (FA) composition (g/100 g of FA) from cows fed CHAM ^1^, CHPM ^1^, and RGWC ^1^.

Item	CHAM	CHPM	RGWC	SEM ^4^	*p*-Value
C4:0:C12:0	17.2	15.8	15.5	0.44	0.406
C14:0	11.5	11.1	11.0	0.3	0.131
C14:0iso	0.09b	0.10b	0.11a	0.004	0.003
C15:0iso	0.28b	0.27b	0.32a	0.007	<0.0001
C15:0	1.19	1.15	1.17	0.025	0.368
C15:0anteiso	0.56	0.56	0.62	0.053	0.589
C16:0	31.9	32.8	32.8	0.67	0.369
C16:0iso	0.23	0.23	0.24	0.007	0.512
C17:0	0.55b	0.55b	0.59a	0.026	0.02
C17:0iso	0.43b	0.44b	0.49a	0.036	0.008
C17:0anteiso	0.56	0.55	0.54	0.014	0.611
C18:0	11.2	11.7	12.3	0.5	0.723
C18:1t11	2.49b	2.43b	3.02a	0.333	0.007
C18.1c9	16.4	17.1	16.7	0.563	0.553
C18:2 c9,12	1.16a	1.21a	0.90b	0.059	<0.0001
C18:3 c9,12,15	1.12a	1.18a	0.94b	0.04	<0.0001
c9 t11 CLA	0.94	0.87	1.10	0.068	0.059
C20:0	0.12	0.11	0.26	0.005	0.0007
C22:0	0.08	0.08	0.08	0.008	0.945
C20:5 c5,8,11,14,17	0.11a	0.10b	0.10b	0.003	0.033
C22:5 c7,10,13,16,19	0.12	0.12	0.11	0.004	0.619
Saturated FA	73.4	72.2	72.4	0.581	0.954
Monounsaturated FA	22.2	23.5	23.7	0.588	0.6
Polyunsaturated FA	4.28a	4.38a	3.9b	0.074	<0.0001
*de novo* ^2^	25.9	24.9	24.7	0.69	0.051
Omega-3	1.35a	1.39a	1.15b	0.042	<0.0001
Omega-6	1.16a	1.21a	0.90b	0.059	<0.0001
Trans	3.74ab	3.51b	4.11a	0.16	0.025
Others ^3^	6.01	6.2	5.3	0.89	0.114

a,b Means within a row with different letters differ (*p* < 0.05). ^1^ RGWC = perennial ryegrass/white clover only; CHAM = ryegrass/white clover + morning allocation of chicory; CHPM = ryegrass/white clover + afternoon allocation of chicory. ^2^ *de novo* includes fatty acids with <16 carbon atoms. ^3^ Others include C14:1c9, C16.1t9, C16.1c7, C16.1c9, C18.1c6, C18.1c11, C18.1c12, C18.1c15, C18.1t9, C18.1t10, C18.2t9c12, C18.2c9t13, C20.1c8. ^4^ SEM = standard error of the mean.

## Supplementary Materials

The following are available online at https://www.mdpi.com/2076-2615/10/1/169/s1.
