# Supplementary material for: Rumen Fermentation and Fatty Acid Composition of Milk of Mid Lactating Dairy Cows Grazing Chicory and Ryegrass
Source: Animals (Basel). 2020 Jan 19;10(1):169. doi: 10.3390/ani10010169 (PMC7023442; doi:10.3390/ani10010169)
Supplement: Supplementary file 1 [file animals-10-00169-s001.docx]

**S1 Table. Post-grazing chemical composition and fatty acid (FA) profile of chicory and ryegrass/white clover (RGWC) herbage sampled to ground level.**

| **Herbage** | **Chicory herbage** | | **Ryegrass/white clover herbage** | | |  | **SEM^2^** | **P-value** | | |
| --- | --- | --- | --- | --- | --- | --- | --- | --- | --- | --- |
| **Treatments** | **CHAM** | **CHPM** |  | **CHAM** | **CHPM** | **RGWC** |  | **Treatment** | **Herbage** | **T x H** |
| OM (g/kg DM) | 859 | 886 |  | 941 | 938 | 923 | 11.1 | 0.27 | <0.001 | 0.213 |
| WSC (g/kg DM) | 108 | 106 |  | 197 | 207 | 191 | 12.7 | 0.08 | <0.001 | 0.644 |
| Crude protein (g/kg DM) | 107 | 98 |  | 98 | 109 | 122 | 4.88 | 0.017 | 0.59 | 0.155 |
| NDF (g/kg DM) | 342 | 435 |  | 573 | 559 | 546 | 21.3 | 0.021 | <0.001 | 0.031 |
| ADF (g/kg DM) | 261 | 301 |  | 327 | 323 | 3.4 | 11.1 | 0.171 | 0.0037 | 0.052 |
| DMD (g/kg DM) | 722 | 673 |  | 646 | 646 | 656 | 14.4 | 0.19 | 0.005 | 0.117 |
| DOMD (g/kg DM) | 652 | 618 |  | 633 | 634 | 636 | 15.9 | 0.5 | 0.937 | 0.299 |

^1^ RGWC = perennial ryegrass/white clover only; CHAM = ryegrass/white clover + morning allocation of chicory; CHPM = ryegrass/white clover + afternoon allocation of chicory. ^2^ SEM = standard error of the mean.

**S2 Table. Volatile fatty acid (VFA) concentration (mmol/L) in ruminal digesta of cannulated dairy cows fed ryegrass and chicory herbage diets.**

| **Sampling time** | **Treatments^1^** | **Acetate** | **Propionate** | **Butyrate** | **Isobutyrate** | **Valerate** | **Isovalerate** | **Hexanoic** | **Total VFA** |
| --- | --- | --- | --- | --- | --- | --- | --- | --- | --- |
| 4h | CHAM | 95 | 24.8 | 17 | 0.98 | 1.45 | 1.31 | 0.303 | 141 |
|  | CHPM | 101 | 24.2 | 16.6 | 0.99 | 1.35 | 1.41 | 0.129 | 145 |
|  | RGWC | 94 | 24.1 | 15.1 | 0.90 | 1.24 | 1.21 | 0.165 | 137 |
| 8h | CHAM | 81 | 18.9 | 13.1 | 0.81 | 1.01 | 1.03 | 0.233 | 116 |
|  | CHPM | 86 | 18.8 | 14.3 | 0.84 | 1.00 | 1.14 | 0.077 | 122 |
|  | RGWC | 84 | 18.8 | 12.5 | 0.87 | 0.95 | 1.18 | 0.135 | 118 |
| 12h | CHAM | 103 | 26.7 | 15.5 | 1.08 | 1.32 | 1.61 | 0.167 | 150 |
|  | CHPM | 89 | 22.1 | 15.7 | 1.29 | 1.52 | 1.92 | 0.149 | 132 |
|  | RGWC | 86 | 20.8 | 13.7 | 1.13 | 1.20 | 1.61 | 0.097 | 125 |
| 16h | CHAM | 99 | 23 | 15.2 | 0.91 | 1.14 | 1.23 | 0.122 | 140 |
|  | CHPM | 84 | 19.2 | 14.5 | 1.14 | 1.19 | 1.57 | 0.106 | 120 |
|  | RGWC | 86 | 20.5 | 14.1 | 1.09 | 1.15 | 1.50 | 0.091 | 124 |
| 20h | CHAM | 106 | 28.2 | 20.9 | 1.26 | 1.74 | 1.95 | 0.240 | 160 |
|  | CHPM | 111 | 29 | 18.6 | 1.26 | 1.60 | 1.96 | 0.175 | 164 |
|  | RGWC | 91 | 22.7 | 16.1 | 0.99 | 1.24 | 1.41 | 0.119 | 134 |
| 24h | CHAM | 90 | 22 | 15.1 | 0.94 | 1.19 | 1.27 | 0.136 | 130 |
|  | CHPM | 96 | 23.3 | 16.4 | 1.07 | 1.36 | 1.49 | 0.113 | 140 |
|  | RGWC | 88 | 21 | 14.5 | 0.94 | 1.10 | 1.25 | 0.117 | 127 |
| SEM^2^ |  | 3.87 | 1.52 | 1.26 | 0.071 | 0.098 | 0.115 | 0.033 | 5.92 |
| P-values | Treatment (Tr) | 0.013 | 0.019 | 0.023 | 0.305 | 0.014 | 0.105 | 0.01 | 0.009 |
|  | Time (Ti) | <0.001 | <0.001 | <0.001 | <0.001 | <0.001 | <0.001 | 0.001 | <0.001 |
|  | Tr × Ti | 0.005 | 0.026 | 0.31 | 0.072 | 0.093 | 0.35 | 0.037 | 0.024 |

^1^ RGWC = perennial ryegrass/white clover only; CHAM = ryegrass/white clover + morning allocation of chicory; CHPM = ryegrass/white clover + afternoon allocation of chicory. ^2^ SEM = standard error of the mean.

**S3 Table. Medium chain fatty acid concentration (g/100 g of FA) in ruminal digesta of cannulated dairy cows fed chicory and ryegrass herbage diets.**

|  |  | **Sampling time** | | | | | |  | **P-values** |  |  |
| --- | --- | --- | --- | --- | --- | --- | --- | --- | --- | --- | --- |
| **Item** | **Treatments^1^** | **4h** | **8h** | **12h** | **16h** | **20h** | **24h** | **SEM^2^** | **Treatment** | **Time** | **Interaction** |
| C12:0 | CHAM | 0.548 | 0.815 | 0.431 | 0.513 | 0.667 | 0.633 |  |  |  |  |
|  | CHPM | 0.712 | 0.711 | 0.535 | 0.595 | 0.471 | 0.56 |  |  |  |  |
|  | RGWC | 1.101 | 0.987 | 0.779 | 0.883 | 0.791 | 0.769 | 0.0793 | 0.001 | 0.0036 | 0.233 |
| C13:0 | CHAM | 0.1056 | 0.1147 | 0.0939 | 0.0831 | 0.0336 | 0.1283 |  |  |  |  |
|  | CHPM | 0.1651 | 0.1646 | 0.0791 | 0.1221 | 0.1103 | 0.1268 |  |  |  |  |
|  | RGWC | 0.1926 | 0.1836 | 0.1287 | 0.1429 | 0.13 | 0.1269 | 0.025 | 0.072 | 0.007 | 0.559 |
| C14:0 | CHAM | 1.46 | 2.24 | 1.39 | 1.65 | 1.9 | 1.72 |  |  |  |  |
|  | CHPM | 2.14 | 2.28 | 1.57 | 1.89 | 1.6 | 1.78 |  |  |  |  |
|  | RGWC | 2.74 | 2.63 | 2.07 | 2.3 | 2.02 | 2.04 | 0.202 | 0.006 | 0.003 | 0.359 |
| isoC14:0 | CHAM | 0.571 | 0.874 | 0.644 | 0.675 | 0.644 | 0.632 |  |  |  |  |
|  | CHPM | 0.797 | 0.98 | 0.644 | 0.767 | 0.561 | 0.654 |  |  |  |  |
|  | RGWC | 0.689 | 0.702 | 0.501 | 0.535 | 0.708 | 0.754 | 0.159 | 0.871 | 0.172 | 0.688 |
| C15:0 | CHAM | 1.75 | 2.75 | 1.75 | 2.12 | 2.12 | 2 |  |  |  |  |
|  | CHPM | 2.5 | 2.72 | 1.84 | 2.26 | 1.73 | 2.05 |  |  |  |  |
|  | RGWC | 2.8 | 2.84 | 2.12 | 2.2 | 1.96 | 2.06 | 0.191 | 0.263 | <.0001 | 0.118 |
| isoC15:0 | CHAM | 1.53 | 2.49 | 1.46 | 1.84 | 1.91 | 1.77 |  |  |  |  |
|  | CHPM | 2.27 | 2.64 | 1.64 | 2.13 | 1.56 | 1.83 |  |  |  |  |
|  | RGWC | 3.07 | 3.16 | 2.18 | 2.4 | 2.07 | 2.12 | 0.227 | 0.006 | 0.0001 | 0.297 |
| anteisoC15:0 | CHAM | 2.99 | 4.9 | 2.95 | 3.52 | 3.72 | 3.5 |  |  |  |  |
|  | CHPM | 4.48 | 4.88 | 2.96 | 3.64 | 2.92 | 3.52 |  |  |  |  |
|  | RGWC | 4.96 | 5.01 | 3.52 | 3.72 | 3.31 | 3.45 | 0.424 | 0.43 | 0.0001 | 0.258 |
| C16:0 | CHAM | 18.9 | 24 | 21.7 | 23.3 | 25.5 | 23 |  |  |  |  |
|  | CHPM | 25.7 | 26.2 | 22.1 | 23.5 | 22.7 | 23.9 |  |  |  |  |
|  | RGWC | 24.3 | 23.7 | 21.9 | 22.3 | 20.8 | 22 | 1.79 | 0.642 | 0.415 | 0.152 |
| isoC16:0 | CHAM | 0.547 | 0.966 | 0.55 | 0.665 | 0.694 | 0.682 |  |  |  |  |
|  | CHPM | 0.775 | 1.073 | 0.643 | 0.814 | 0.567 | 0.66 |  |  |  |  |
|  | RGWC | 1.002 | 1.1 | 0.813 | 0.832 | 0.764 | 0.789 | 0.078 | 0.015 | 0.0001 | 0.317 |
| C16:1 c9 | CHAM | 0.164 | 0.217 | 0.145 | 0.103 | 0.113 | 0.196 |  |  |  |  |
|  | CHPM | 0.259 | 0.255 | 0.203 | 0.206 | 0.201 | 0.2 |  |  |  |  |
|  | RGWC | 0.204 | 0.182 | 0.218 | 0.279 | 0.281 | 0.351 | 0.049 | 0.143 | 0.564 | 0.191 |
| C17:0 | CHAM | 0.353 | 0.41 | 0.301 | 0.331 | 0.329 | 0.338 |  |  |  |  |
|  | CHPM | 0.385 | 0.413 | 0.369 | 0.392 | 0.292 | 0.324 |  |  |  |  |
|  | RGWC | 0.439 | 0.477 | 0.409 | 0.415 | 0.411 | 0.443 | 0.019 | 0.0203 | 0.0001 | 0.0001 |
| isoC17:0 | CHAM | 0.294 | 0.297 | 0.296 | 0.328 | 0.333 | 0.318 |  |  |  |  |
|  | CHPM | 0.357 | 0.505 | 0.358 | 0.446 | 0.285 | 0.314 |  |  |  |  |
|  | RGWC | 0.476 | 0.541 | 0.443 | 0.436 | 0.396 | 0.421 | 0.045 | 0.005 | 0.062 | 0.302 |
| anteisoC17:0 | CHAM | 0.393 | 0.709 | 0.435 | 0.53 | 0.528 | 0.481 |  |  |  |  |
|  | CHPM | 0.558 | 0.9 | 0.566 | 0.759 | 0.467 | 0.476 |  |  |  |  |
|  | RGWC | 0.707 | 0.848 | 0.664 | 0.642 | 0.585 | 0.603 | 0.069 | 0.029 | 0.0001 | 0.313 |

^1^ RGWC = perennial ryegrass/white clover only; CHAM = ryegrass/white clover + morning allocation of chicory; CHPM = ryegrass/white clover + afternoon allocation of chicory. ^2^ SEM = standard error of the mean.

**S4 Table. Long chain fatty acid concentration (g/100 g of FA) in ruminal digesta of cannulated dairy cows fed chicory and ryegrass herbage diets.**

|  |  |  | **Time** |  |  |  |  |  | **P-value** |  |  |
| --- | --- | --- | --- | --- | --- | --- | --- | --- | --- | --- | --- |
| **Item** | **Treatment** | **4h** | **8h** | **12h** | **16h** | **20h** | **24h** | **SEM** | **Treatment** | **Time** | **Interaction** |
| C18:0 | CHAM | 26.2 | 15.6 | 14.6 | 15.4 | 12.6 | 17.8 |  |  |  |  |
|  | CHPM | 13.5 | 14.7 | 17.5 | 19.7 | 10.9 | 13.5 |  |  |  |  |
|  | RGWC | 12.9 | 14.2 | 15 | 14 | 17.6 | 18.7 | 3.38 | 0.725 | 0.707 | 0.128 |
| C18:1, t9 | CHAM | 0.177 | 0.214 | 0.256 | 0.219 | 0.241 | 0.267 |  |  |  |  |
|  | CHPM | 0.197 | 0.121 | 0.191 | 0.103 | 0.234 | 0.214 |  |  |  |  |
|  | RGWC | 0.11 | 0.114 | 0.184 | 0.124 | 0.142 | 0.116 | 0.07 | 0.498 | 0.381 | 0.901 |
| C18:1 t11 | CHAM | 3.78 | 4.39 | 3.67 | 3.96 | 4.35 | 4.22 |  |  |  |  |
|  | CHPM | 4.01 | 4.98 | 4.65 | 4.95 | 3.35 | 3.39 |  |  |  |  |
|  | RGWC | 6.03 | 4.07 | 4.2 | 4.01 | 4.75 | 4.82 | 0.352 | 0.11 | 0.446 | 0.0003 |
| C18:1 c9 | CHAM | 3.66 | 3.93 | 4.17 | 4.34 | 4.57 | 4.29 |  |  |  |  |
|  | CHPM | 4.43 | 4.37 | 4.18 | 3.96 | 4.59 | 4.53 |  |  |  |  |
|  | RGWC | 4.04 | 4.07 | 4.13 | 3.98 | 3.98 | 4.05 | 0.26 | 0.603 | 0.182 | 0.092 |
| C18:1 c11 | CHAM | 0.606 | 0.77 | 0.59 | 0.653 | 0.741 | 0.682 |  |  |  |  |
|  | CHPM | 0.886 | 0.838 | 0.668 | 0.706 | 0.697 | 0.767 |  |  |  |  |
|  | RGWC | 0.863 | 0.786 | 0.694 | 0.718 | 0.673 | 0.688 | 0.07 | 0.392 | 0.046 | 0.376 |
| C18:2 t9,12 | CHAM | 0.575 | 0.865 | 0.452 | 0.542 | 0.303 | 0.386 |  |  |  |  |
|  | CHPM | 0.463 | 0.338 | 0.351 | 0.376 | 0.293 | 0.253 |  |  |  |  |
|  | RGWC | 0.428 | 0.909 | 0.526 | 0.705 | 0.444 | 0.311 | 0.182 | 0.429 | 0.058 | 0.772 |
| C18:2 c9,12 | CHAM | 4.55 | 5.87 | 8.93 | 8.43 | 7.83 | 6.34 |  |  |  |  |
|  | CHPM | 8.17 | 6.79 | 6.53 | 6.05 | 10.37 | 8.33 |  |  |  |  |
|  | RGWC | 5.87 | 5.66 | 6.8 | 6.24 | 5.87 | 6.04 | 0.86 | 0.249 | 0.011 | 0.0007 |
| C18:3 c9,12,15 | CHAM | 3.82 | 4.9 | 12.83 | 8.26 | 9.01 | 6.82 |  |  |  |  |
|  | CHPM | 6.32 | 3.72 | 8.36 | 5.06 | 14.12 | 8.99 |  |  |  |  |
|  | RGWC | 4.93 | 4.38 | 8.14 | 6.58 | 5.88 | 6.77 | 1.51 | 0.534 | 0.0001 | 0.002 |
| C19:0 | CHAM | 1.34 | 1.11 | 1.41 | 1.24 | 1.61 | 1.07 |  |  |  |  |
|  | CHPM | 1.33 | 1.38 | 1.66 | 1.35 | 1.61 | 1.42 |  |  |  |  |
|  | RGWC | 1.05 | 1.04 | 1.34 | 1.3 | 1.34 | 1.3 | 0.19 | 0.132 | 0.076 | 0.867 |
|  | CHAM | 0.591 | 0.663 | 0.497 | 0.52 | 0.565 | 0.538 |  |  |  |  |
|  | CHPM | 0.577 | 0.54 | 0.557 | 0.588 | 0.485 | 0.554 |  |  |  |  |
|  | RGWC | 0.797 | 0.728 | 0.707 | 0.77 | 0.635 | 0.699 | 0.043 | 0.008 | 0.023 | 0.131 |

^1^RGWC = perennial ryegrass/white clover only; CHAM = ryegrass/white clover + morning allocation of chicory; CHPM = ryegrass/white clover + afternoon allocation of chicory.

^2^SEM = standard error of the mean.
